# Weight Loss Medications in Young Adults after Bariatric Surgery for Weight Regain or Inadequate Weight Loss: A Multi-Center Study

**DOI:** 10.3390/children5090116

**Published:** 2018-08-29

**Authors:** Alexander T. Toth, Gricelda Gomez, Alpana P. Shukla, Janey S. Pratt, Hellas Cena, Ginevra Biino, Louis J. Aronne, Fatima Cody Stanford

**Affiliations:** 1 Adolescent Neuroendocrine Unit, Massachusetts General Hospital, Boston, MA 02114, USA; attoth@mgh.harvard.edu; 2 Department of Surgery-Urology, Brigham and Women’s Hospital, Boston, MA 02115, USA; ggomez@bwh.harvard.edu; 3 Harvard Medical School, Boston, MA 02115, USA; 4 Comprehensive Weight Control Center, Division of Endocrinology, Diabetes, and Metabolism, Department of Medicine, Weill Cornell Medical College, New York, NY 10065, USA; aps2004@med.cornell.edu (A.P.S.); ljaronne@med.cornell.edu (L.J.A.); 5 Stanford University School of Medicine, Stanford, CA 94305, USA; jsapratt@stanford.edu; 6 Lucile Packard Children’s Hospital, Stanford, CA 94304, USA; 7 Laboratory of Dietetics and Clinical Nutrition, Department of Public Health, Experimental and Forensic Medicine, University of Pavia, 27100 Pavia, Italy; hellas.cena@unipv.it; 8 Institute of Molecular Genetics, National Research Council of Italy, 27100 Pavia, Italy; biino@igm.cnr.it; 9 Department of Pediatrics-Endocrinology, Massachusetts General Hospital, Boston, MA 02114, USA; 10 Department of Medicine-Neuroendocrine Unit, Boston, MA 02114, USA

**Keywords:** obesity, bariatric surgery, weight loss surgery, young adults, weight loss medications, anti-obesity pharmacotherapy, weight regain

## Abstract

This paper presents a retrospective cohort study of weight loss medications in young adults aged 21 to 30 following Roux-en-Y gastric bypass (RYGB) and sleeve gastrectomy (SG) between November 2000 and June 2014. Data were collected from patients who used topiramate, phentermine, and/or metformin postoperatively. Percentage of patients achieving ≥5%, ≥10%, or ≥15% weight loss on medications was determined and percent weight change on each medication was compared to percent weight change of the rest of the cohort. Our results showed that 54.1% of study patients lost ≥5% of their postsurgical weight; 34.3% and 22.9% lost ≥10% and ≥15%, respectively. RYGB had higher median percent weight loss (−8.1%) than SG (−3.3%) (*p* = 0.0515). No difference was found in median percent weight loss with medications started at weight plateau (−6.0%) versus after weight regain (−5.4%) (*p* = 0.5304). Patients taking medications at weight loss plateau lost 41.2% of total body weight from before surgery versus 27.1% after weight regain (*p* = 0.076). Median percent weight change on metformin was −2.9% compared to the rest of the cohort at −7.7% (*p* = 0.0241). No difference from the rest of the cohort was found for phentermine (*p* = 0.2018) or topiramate (*p* = 0.3187). Topiramate, phentermine, and metformin are promising weight loss medications for 21 to 30 year olds. RYGB patients achieve more weight loss on medications but both RYGB and SG benefit. Median total body weight loss from pre-surgical weight may be higher in patients that start medication at postsurgical nadir weight. Participants on metformin lost significantly smaller percentages of weight on medications, which could be the result of underlying medical conditions.

## 1. Introduction

Bariatric surgery is the most effective treatment for moderate obesity (body mass index (BMI) 35–39.9) and severe obesity (BMI ≥ 40) [1,2,3]. Following surgery, patients often experience complete or partial resolution of obesity-related co-morbidities with weight loss, but may struggle with inadequate weight loss or weight regain [4]. Unfortunately, co-morbidities often return with weight regain [5,6]. Surgical revisions may be attempted and have occasionally demonstrated improvements in obesity co-morbidities and weight loss, however, the risk of complications with revision procedures is significantly higher than with initial bariatric surgery [7,8]. While alternative surgical solutions for weight regain or inadequate weight loss exist, they do not prove to be sufficient long-term solutions [9,10]. Additionally, studies have shown that significant weight regain occurs in 25–35% of people who have bariatric surgery within 2–5 years of their procedure [11,12].

Weight regain, and inadequate weight loss, are problems that require additional therapies other than surgery, for which medications provide a promising adjuvant therapy.

Multiple studies have evaluated medications for use in cases where inadequate weight loss or weight regain occur in adults across the age spectrum [9,10,13,14,15,16]. Stanford and colleagues demonstrated that weight loss medications were a useful tool to confer additional weight loss in a study containing a wide age range of adult patients after Roux-en-Y gastric bypass (RYGB) and sleeve gastrectomy (SG), the two most common weight loss surgeries performed currently in the United States [17]. In this study, we use the same data set as Stanford and colleagues to explore the utility of three common weight loss medications in a younger subset of patients who had surgery between 21 and 30 years of age.

It is important to consider the possibility of varying responses to medications in young adults due to physiologic changes that occur with aging and the influence these changes have on both pharmacokinetics and pharmacodynamics. Differences in responses to both psychotropic and cardiovascular drugs have been observed in older adults compared to younger adults [18]. It is unknown whether differences in response to weight loss medications exist in younger and older adults [19]. Our goal is to demonstrate the utility of common weight loss medications in younger adults who have inadequate weight loss or regain after bariatric surgery and to show that young adults respond to weight loss medication therapy in a similar fashion to patients across the age spectrum.

## 2. Materials and Methods

### 2.1. Sample and Data Collection

The patients in our study sample came from two major academic medical centers where they underwent Roux-en-Y gastric bypass (RYGB) or sleeve gastrectomy (SG) procedures between November 2000 and June 2014. From our sample, all patients who were 30 years or younger that were subsequently placed on topiramate, phentermine, or metformin after surgery were considered for inclusion. All patients had at least 12 months of documented postoperative follow up. Patients were excluded if they did not have sufficient follow up, had additional surgery for complications within 6 months of their initial surgery, or if they required a revision surgery. Two research groups obtained the clinical data from the medical record and the project was approved by the institutional review boards at both academic centers.

### 2.2. Demographic and Clinical Factors

We obtained the following data from the medical records of eligible patients: (1) type of surgery (RYGB or SG); (2) date of operation; (3) date of birth; (4) gender; (5) race/ethnicity (Caucasian, Hispanic, Black, Asian, or other); (6) preoperative obesity-related co-morbidities (hypertension, type 2 diabetes, obstructive sleep apnea (OSA), dyslipidemia, and non-alcoholic fatty liver disease (NAFLD)); (7) preoperative use of weight loss medications; (8) BMI (based on initial height and weight at the following time points: pre-surgery, at plateau post-surgery, at the start of weight loss medication, at plateau post-weight loss medication, and current; (9) time to achieve plateau weight post-surgery.

### 2.3. Weight Loss Medications

We collected information on three medications that are prescribed by obesity medicine physicians at the study staff’s clinics. These medications were: (1) phentermine, (2) topiramate, and (3) metformin. Medications were started at different times following surgery. The statistical analysis includes testing for differences in percent weight loss based on whether medications were given at plateau weight or after weight regain. Plateau weight was defined as having a weight within 3% of the nadir weight achieved following surgery. Patients whose weight was not within 3% of the nadir weight achieved following surgery, at the time of medication start, were considered to have started medication after weight regain.

### 2.4. Primary Endpoints

We had four coprimary endpoints captured at the weight plateau after medication administration. These endpoints were: (1) percentage change in weight on medications; (2) the proportion of patients losing at least 5% of postsurgical weight; (3) the proportion of patients losing at least 10% of postsurgical weight; and (4) the proportion of patients losing at least 15% of postsurgical weight.

### 2.5. Statistical Analysis

We ascertained patient weight histories, including nadir weight post-surgery but before weight loss medication treatment, weight at initiation of weight loss medication treatment, and weight at new nadir following weight loss medication treatment to determine percent change in weight on medications. We evaluated these parameters with descriptive statistics and evaluated our cohort by surgery type (RYGB or SG). Baseline demographic characteristics and preoperative baseline characteristics were also included. The treatment period of medications was determined by the time between the date weight loss medications were initiated to the date when new nadir weight on medications was achieved or by the length of the total treatment period if a new nadir weight was not achieved.

Shapiro-Wilk test of normality was performed to determine that data was non-parametric and non-parametric statistical methods were utilized to test for significance. The Mann Whitney test was used to determine whether percent weight change achieved with medications in the RYGB and SG groups were significantly different. The same test was employed to test for difference in percent weight loss between participants who had medications prescribed at post-op weight nadir or after weight regain. The Mann Whitney test was also used to determine whether differences in median percent weight change achieved while taking each medication were significantly different compared to median percent weight change of study participants who did not receive the same medication. Pearson rho testing was used to evaluate correlation between BMI at medication start and percent weight loss achieved on medications.

## 3. Results

### 3.1. Participants

Baseline characteristics of study subjects are noted in Table 1. Of the 5110 patient records that were reviewed, 37 (0.7%) met criteria for inclusion. Patients were predominantly female (*n* = 37; 90.2%) and were between the ages of 21 and 30 years old, with 15 (40.5%) patients between 21 and 25 years old and 22 (59.5%) patients between 26 and 30 years old. There were 24 (64.9%) patients who identified as Caucasian, 7 (18.9%) identified as Hispanic, 5 (13.5%) identified as African-American, and 1 (3.6%) declined to answer or identified as a different race. At the time of surgery, RYGB patients had higher mean BMI (51.7 kg/m^2^; standard deviation (SD) = 11.1) versus SG patients (47.1 kg/m^2^; SD = 6), higher percentage of obesity related co-morbidities, and took longer to reach their weight plateau after surgery. All but four patients achieved greater than 20% of total body weight loss, which has been used as a weight loss metric for success of bariatric surgery [20]. At the start of medication, as denoted in Table 2, the mean BMI of RYGB (BMI = 38.9 kg/m^2^; SD = 9.1) and SG (BMI = 37.3 kg/m^2^; SD = 5.7) were similar, but RYGB patients had a longer time elapse between surgery (62.6 months; SD = 39.1) and the start of medication compared with SG patients (20.1 months; SD = 5.2). At the nadir weight achieved post-weight loss medication, the mean BMI for patients was similar between the RYGB (BMI = 33.4 kg/m^2^; SD = 6.6) and SG (BMI = 35.9 kg/m^2^; SD = 5.6) groups.

**Figure 1:**
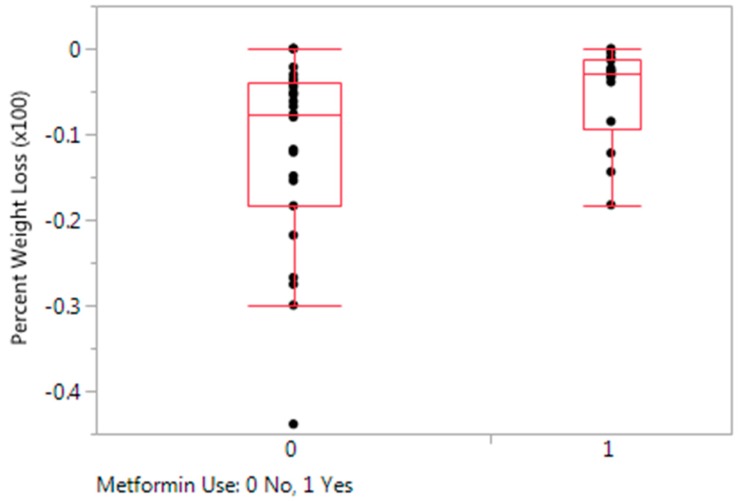
Percent Weight Loss with and without Metformin. Participants on metformin had a significant difference in median percent weight change on medications (−2.9%) compared to the rest of the cohort (−7.7%) (*p* = 0.0241).

Patients that were prescribed Metformin had a median percent weight loss on medications (−2.9%) that was significantly lower than the median percent weight loss of the rest of the study cohort (−7.7%) (*p* = 0.0241) (Figure 1). No significant difference in median percent weight loss on medications was found between those participants on phentermine (−7.7%) and the rest of the study cohort (−3.1%) (*p =* 0.2018). No significant difference in median percent weight loss on medications was found between those participants on topiramate (−7.2%) and the rest of the study cohort (−4.4%) (*p =* 0.3187).

### 3.2. Weight Loss Medications and Response

More than half (54.1%; *n* = 20) of all patients lost ≥5% of their postsurgical weight with treatment. There were also high responders to medications, with 34.5% of patients (*n* = 12) and 22.9% (*n* = 8) losing ≥10% and ≥15% of postsurgical weight, respectively (Table 3). Patients in the RYGB group achieved a greater percent weight change on medications (−8.1%, interquartile range (IQR): −2.6%, −17.5%) compared to SG patients (−3.3%, IQR: −2.6%, −17.5%), with the differences approaching statistical significance (*p* = 0.0515) (Table 3). BMI at the start of medications was not found to be statistically correlated with percent weight loss on medications in this cohort (*r* = 0.2482, *p* = 0.1386). Patients were more likely to be prescribed medications after weight regain (78.4%; *n* = 29) had occurred than at their plateau (21.6%; *n* = 8) (Table 3). No statistical difference was found in percent weight loss on medications when medications were prescribed at weight regain (−5.4%) or at weight plateau (−6.0%) (*p* = 0.5304). Patients prescribed weight loss medications at weight plateau lost a greater percentage (−41.2%) of total body weight from pre-surgery to their new nadir weight following medications compared to patients who started medications after regain (−27.1%) (*p* = 0.0766) (Table 3).

## 4. Discussion

In this study, 54.1%, 34.3%, and 22.9% of patients achieved ≥5%, ≥10%, and ≥15% additional weight loss, respectively, which was very similar to results found by Stanford and colleagues’ study on medication use for additional weight loss after RYGB and SG, which demonstrated that 56%, 30%, and 16% of the patients it included achieved ≥5%, ≥10%, and ≥15% additional weight loss, respectively [17]. In the prior study, patients were between the ages of 21 and 73 years old [17]. We saw similar results in our younger study population, which is important because age is a predictor of weight regain after surgery [20]. Even modest weight reductions can lower risk of, or improve, cardiovascular disease, liver disease, and diabetes. Our confirmation that medication therapy can help confer additional weight loss after bariatric surgery in this population supports the use of pharmacotherapy in young adults. The use of weight loss medications has been shown to help patients across a broad range of ages lose additional weight after bariatric procedures [9,10,13,14,15,16,17]. Our study confirmed this for patients between the ages of 21 and 30 who had RYGB and SG. The median weight loss achieved with medications was −5.1% (−5.8 kg).

A recent 2018 study by Nor Hanipah and colleagues found a significant positive correlation between BMI at medication start and weight loss [14]. We found a non-significant slightly positive correlation between percent weight loss and BMI at the start of medications (*r* = 0.2482, *p* = 0.1386). This lack of correlation may be due to our small sample size. We also did not find a statistically significant difference in percentage of weight lost when medications were prescribed at weight plateau versus once weight regain had occurred, which agrees with previous findings on weight loss medication use post-surgery [9,10,13,14,15,16,17]. We chose to set a cut off to define patients either as part of a weight plateau group versus a weight regain group. A recent follow-up study by Ryder and colleagues explored factors associated with long-term weight-loss maintenance following bariatric surgery in adolescents at 5 years following surgery [21]. Ryder and colleagues used a cut off of 20% at 5 years to group patients that had maintained postsurgical weight loss or regained weight [21]. We grouped patients similarly but chose to use a smaller cutoff of 3% for this application to explore whether there was a difference in percent weight loss achieved on medications in patients able to stay closer to their nadir weight compared to those who started to regain more than 3% of their nadir weight. We did not find a significant difference in percent weight loss between these two groups but found a difference that seemed to approach significance in percent of total body weight lost from pre-surgical weight by patients who started medications at their post-operation nadir (−41.2%) compared to patients who started medications after weight regain (−27.1%) (*p* = 0.0766). Further research needs to be performed into the timing of weight loss medication, but our results may support prescribing medication at the nadir weight post-surgery as the best strategy to help patients achieve the greatest possible total weight loss [17].

Between the two surgery types studied, we found a difference in percent weight loss achieved on medications that approached statistical significance. RYGB participants lost a median of 8.1% of their weight on medications while SG participants lost a median of 3.3% of their weight on medications (*p* = 0.0515). This finding agrees with recent findings by Nor Hanipah and colleagues that found response to weight loss medications to be significantly better in RYGB and gastric banding patients compared to SG patients [14]. It has also been previously observed that RYGB patients have a higher likelihood of weight loss compared to patients who undergo SG [17,22]. Differences in the two surgeries and the physiologic changes that occur in the two may explain varying responses to different weight loss medications [23].

In the previous study using the same data set by Stanford and colleagues that evaluated patients aged 21 to 73 years old, a statistically significant increase in likelihood of additional weight loss after bariatric surgery with topiramate was observed [17]. In this study of the same data, which focused solely on 21 to 30 year olds, we did not observe a statistically significant difference in percent weight loss on topiramate or phentermine from the rest of the cohort, but we did find that patients using metformin had a statistically lower percentage of weight loss on medications compared to the rest of the cohort. Metformin is a well-tested, safe drug for use in diabetes treatment and for the prevention of diabetes in cases of pre-diabetes [24]. Metformin has shown success in trials for weight loss in patients without diabetes [25] and may decrease appetite and oppose unfavorable fat storage in peripheral tissues [26]. Unfortunately, the data available in this study do not allow for us to evaluate the indication for which metformin was prescribed in each case. A likely explanation for the lower percentage weight loss on metformin is that medical conditions often treated by metformin such as insulin resistance, type 2 diabetes, and metabolic syndrome can make weight loss more difficult. The finding that patients lost a lower percentage of weight on metformin is not an indictment of metformin as a treatment but is an interesting result that needs further research to understand.

There are many limitations to our study due to its retrospective nature. The largest limitation being a smaller sample size due to a smaller available data set for the younger adults between the ages of 21–30. Other limitations include missing patient data, no control group, inability to account for the length of time that patients were on weight loss medications, confounding factors including concurrent treatment with weight promoting medications, two different surgery types, and weight loss medications being evaluated for weight loss potential when they may have been prescribed for other indications. We were also unable to measure the effect of diet and exercise nor account for some medications prescribed more frequently than others. Despite these limitations, our study data came from two large academic study sites performing the two most common weight loss procedures in the United States and included a long duration of follow up, which gave us insight into the long-term successes achievable in young adults using weight loss medication after bariatric surgery.

## 5. Conclusions

We found that weight loss medications are beneficial for additional weight loss in patients between the ages of 21 and 30 who have undergone bariatric surgery. These results are of particular importance in the long-term treatment of young adults who undergo weight loss surgery. The ideal time to start weight loss medications appears to be at the postsurgical nadir weight. Patients who had RYGB lost a larger percent of weight with medications than SG patients, however, members of both RYGB and SG groups had benefits from the use of medication after bariatric surgery. Further research is needed to explore why patients on metformin lost a lower percentage of weight than other participants, but it is possible that this observed result may be due to underlying medical conditions that make weight loss more difficult.

## Figures and Tables

**Table 1:** Demographic Data and Baseline Characteristics for Patients ≤30 Years Old.

Variable	Surgery Type		
	All Patients	Sleeve Gastrectomy	Roux-En-Y Gastric Bypass
	*n* = 37	*n* = 9 (25.7%)	*n* = 28 (75.7%)
**Gender**			
Female	34 (91.9%)	8 (88.9%)	26 (92.9%)
Male	3 (8.1%)	1 (11.1%)	2 (7.1%)
**Age at Surgery (Years)**			
≤20	0		
21–25	15 (40.5%)	3 (33.3%)	12 (42.9%)
26+	22 (59.5%)	6 (66.7%)	16 (57.1%)
**Race/Ethnicity**			
White	24 (64.9%)	6 (66.7%)	18 (64.3%)
Hispanic	7 (18.9%)	0	7 (25%)
African-American	5 (13.5%)	3 (33.3%)	2 (7.1%)
Asian	0	0	0
Other/Declined to state	1 (2.7%)	0	1 (3.6%)
**Preoperative Characteristics**			
Mean BMI (kg/m^2^)	50.6 (SD = 10.2)	47.1 (SD = 5.8)	51.7 (SD = 11.1)
**Obesity Class**			
Class I (BMI 30–34.9)	0	0	0
Class II (BMI 35–39.9)	5 (13.5%)	1 (11.1%)	4 (14.3%)
Class III (BMI ≥ 40)	32 (86.5%)	8 (88.9%)	24 (85.7%)
**Comorbid Conditions (Individual)**			
Hypertension	10 (27%)	2 (22.2%)	8 (28.6%)
Type II Diabetes	2 (5.4%)	0	2 (7.1%)
OSA	10 (27%)	2 (22.2%)	8 (28.6%)
Dyslipidemia	10 (27%)	3 (33.3%)	7 (25%)
NAFLD	28 (74.3%)	4 (44.4%)	24 (85.7%)
**Number of Comorbid Conditions**			
None	4	2	2
1	16	4	12
2	10	2	8
3	4	1	3
4	3	0	3

BMI—Body Mass Index, OSA—Obstructive Sleep Apnea, NAFLD—Non-Alcoholic Fatty Liver Disease.

**Table 2:** Post-Operative Patient Characteristics and BMI History after Surgery, before Medications, and after Treatment.

	All Patients	Sleeve Gastrectomy	Roux-En-Y Gastric Bypass
Post-Op Nadir Before Medication	*n* = 37	*n* = 9 (24.3%)	*n* = 28 (75.7%)
Mean BMI (kg/m^2^)	33.9 (SD = 7.8)	34.6 (SD = 4.9)	33.7 (SD = 8.6)
Mean Time to Achieve Nadir (months)	17.3 (SD = 11.2)	15.9 (SD = 10.8)	17.7 (SD = 11.4)
At Start of Medication			
Mean BMI (kg/m^2^)	38.5 (SD = 8.3)	37.3 (SD = 5.7)	38.9 (SD = 9.1)
Time elapsed between surgery and start of medication (months)			
Mean (SD)	52.2 (SD = 38.7)	20.1 (SD = 5.2)	62.6 (SD = 39.1)
Min	6.6	9.8	6.6
Max	164.8	28.1	164.8
Post-Medication Treatment—At Nadir Weight			
Mean BMI (kg/m^2^)	34.0 (SD = 6.4)	35.9 (SD = 5.6)	33.4 (SD = 6.6)

**Table 3:** Median Weight Change after Treatment by Subgroup.

Subgroup	Weight Change		*p* Value
	(kg)	(%) ^	
All patients (*n* = 37)	−5.8	−5.1	
	(IQR: −2.5, −16.1)	(IQR: −2.6, −14.6)	
Patients prescribed medication at weight plateau (*n* = 8) ~	−5.7	−6.0	0.5304 ^a^
	(IQR: −3.2, −14.2)	(IQR: −3.1, −20.1))	
Patients prescribed medication at weight regain (*n* = 29) ~	−5.8	−5.4	
	(IQR: −2.3, −16.7)	(IQR: −2.4, −13.3)	
Surgery Type			
Sleeve Gastrectomy (*n* = 9)	−3.9	−3.3	0.0515 ^a^
	(IQR: −1.5, −6.1)	(IQR: −1.5, −5.3)	
Roux-En-Y Gastric Bypass (*n* = 26)	−7.6	−8.1	
	(IQR: −2.4, −17.9)	(IQR: −2.6, −17.5)	
Patients who lost ≥5% total body weight with treatment (*n* = 20, 54.1%)	−15.6	−13.3	
	(IQR: −7.3, −24.3)	(IQR: −7.7, −20.9)	
Patients who lost ≥10% total body weight with treatment (*n* = 12, 34.3%)	−18.1	−18.2	
	(IQR: −15.6, −29.5)	(IQR: −13.3, −27.1)	
Patients who lost ≥15% total body weight with treatment (*n* = 8, 22.9%)	−26.0	−24.3	
	(IQR: −16.1, −34.4)	(IQR: −18.3, −29.4)	

~ Plateau defined as weight that is within 3% above or below nadir weight postoperatively before medication. If above 3%, patient defined as starting medication at weight regain; ^ Calculated this number as ((weight at nadir post-medications) − (weight at start of medication))/(weight at start of medication); ^a^ Mann-Whitney-Wilcoxon test of percent weight change on medication.

## References

[1] Buchwald H., Avidor Y., Braunwald E., Jensen M.D., Pories W., Fahrbach K., Schoelles K. (2004). Bariatric surgery: A systematic review and meta-analysis. JAMA.

[2] Sjostrom L., Peltonen M., Jacobson P., Ahlin S., Andersson-Assarsson J., Anveden A., Bouchard C., Carlsson B., Karason K., Lonroth H. (2014). Association of bariatric surgery with long-term remission of type 2 diabetes and with microvascular and macrovascular complications. JAMA.

[3] Chang S.H., Stoll C.R., Song J., Varela J.E., Eagon C.J., Colditz G.A. (2014). The effectiveness and risks of bariatric surgery: An updated systematic review and meta-analysis, 2003–2012. JAMA Surg..

[4] Mann J.P., Jakes A.D., Hayden J.D., Barth J.H. (2015). Systematic review of definitions of failure in revisional bariatric surgery. Obes. Surg..

[5] DiGiorgi M., Rosen D.J., Choi J.J., Milone L., Schrope B., Olivero-Rivera L., Restuccia N., Yuen S., Fisk M., Inabnet W.B. (2010). Re-emergence of diabetes after gastric bypass in patients with mid- to long-term follow-up. Surg. Obes. Relat. Dis..

[6] Laurino Neto R.M., Herbella F.A., Tauil R.M., Silva F.S., de Lima S.E. (2012). Comorbidities remission after roux-en-y gastric bypass for morbid obesity is sustained in a long-term follow-up and correlates with weight regain. Obes. Surg..

[7] Brethauer S.A., Kothari S., Sudan R., Williams B., English W.J., Brengman M., Kurian M., Hutter M., Stegemann L., Kallies K. (2014). Systematic review on reoperative bariatric surgery: American society for metabolic and bariatric surgery revision task force. Surg. Obes. Relat. Dis..

[8] Fulton C., Sheppard C., Birch D., Karmali S., de Gara C. (2017). A comparison of revisional and primary bariatric surgery. Can. J. Surg..

[9] Zilberstein B., Pajecki D., Garcia de Brito A.C., Gallafrio S.T., Eshkenazy R., Andrade C.G. (2004). Topiramate after adjustable gastric banding in patients with binge eating and difficulty losing weight. Obes. Surg..

[10] Pajecki D., Halpern A., Cercato C., Mancini M., de Cleva R., Santo M.A. (2013). Short-term use of liraglutide in the management of patients with weight regain after bariatric surgery. Rev. Col. Bras. Cir..

[11] Bastos E.C., Barbosa E.M., Soriano G.M., dos Santos E.A., Vasconcelos S.M. (2013). Determinants of weight regain after bariatric surgery. Arq. Bras. Cir. Dig..

[12] Cooper T.C., Simmons E.B., Webb K., Burns J.L., Kushner R.F. (2015). Trends in weight regain following roux-en-y gastric bypass (RYGB) bariatric surgery. Obes. Surg..

[13] Srivastava G., Buffington C. (2018). A specialized medical management program to address post-operative weight regain in bariatric patients. Obes. Surg..

[14] Nor Hanipah Z., Nasr E.C., Bucak E., Schauer P.R., Aminian A., Brethauer S.A., Cetin D. (2018). Efficacy of adjuvant weight loss medication after bariatric surgery. Surg. Obes. Relat. Dis..

[15] Schwartz J., Suzo A., Wehr A.M., Foreman K.S., Mikami D.J., Needleman B.J., Noria S.F. (2016). Pharmacotherapy in conjunction with a diet and exercise program for the treatment of weight recidivism or weight loss plateau post-bariatric surgery: A retrospective review. Obes. Surg..

[16] Jester L., Wittgrove A.C., Clark W. (1996). Adjunctive use of appetite suppressant medications for improved weight management in bariatric surgical patients. Obes. Surg..

[17] Stanford F.C., Alfaris N., Gomez G., Ricks E.T., Shukla A.P., Corey K.E., Pratt J.S., Pomp A., Rubino F., Aronne L.J. (2017). The utility of weight loss medications after bariatric surgery for weight regain or inadequate weight loss: A multi-center study. Surg. Obes. Relat. Dis..

[18] Trifiro G., Spina E. (2011). Age-related changes in pharmacodynamics: Focus on drugs acting on central nervous and cardiovascular systems. Curr. Drug Metab..

[19] Corcelles R., Boules M., Froylich D., Hag A., Daigle C.R., Aminian A., Brethauer S.A., Burguera B., Schauer P.D. (2016). Total weight loss as the outcome measure of choice after roux-en-y gastric bypass. Obes. Surg..

[20] Shantavasinkul P.C., Omotosho P., Corsino L., Portenier D., Torquati A. (2016). Predictors of weight regain in patients who underwent roux-en-y gastric bypass surgery. Surg. Obes. Relat. Dis..

[21] Ryder J.R., Gross A.C., Fox C.K., Kaizer A.M., Rudser K.D., Jenkins T.M., Ratcliff M.B., Kelly A.S., Kirk S., Siegel R.M. (2018). Factors associated with long-term weight-loss maintenance following bariatric surgery in adolescents with severe obesity. Int. J. Obes..

[22] Padwal R., Klarenbach S., Wiebe N., Hazel M., Birch D., Karmali S., Sharma A.M., Manns B., Tonelli M. (2011). Bariatric surgery: A systematic review of the clinical and economic evidence. J. Gen. Intern. Med..

[23] Harvey E.J., Arroyo K., Korner J., Inabnet W.B. (2010). Hormone changes affecting energy homeostasis after metabolic surgery. Mt. Sinai J. Med..

[24] Aroda V.R., Knowler W.C., Crandall J.P., Perreault L., Edelstein S.L., Jeffries S.L., Molitch M.E., Pi-Sunyer X., Darwin C., Heckman-Stoddard B.M. (2017). Metformin for diabetes prevention: Insights gained from the diabetes prevention program/diabetes prevention program outcomes study. Diabetologia.

[25] Seifarth C., Schehler B., Schneider H.J. (2013). Effectiveness of metformin on weight loss in non-diabetic individuals with obesity. Exp. Clin. Endocrinol. Diabetes.

[26] Malin S.K., Kashyap S.R. (2014). Effects of metformin on weight loss: Potential mechanisms. Curr. Opin. Endocrinol. Diabetes Obes..

